# Primary screening for increased fracture risk by the FRAX® questionnaire—uptake rates in relation to invitation method

**DOI:** 10.1007/s11657-019-0603-4

**Published:** 2019-05-08

**Authors:** Louise M. E. Moberg, Peter M. Nilsson, Anna H. Holmberg, Göran Samsioe, Christer Borgfeldt

**Affiliations:** 1 Department of Obstetrics and Gynaecology, Department of Clinical Sciences, Skåne University Hospital, Lund University, SE-221 85 Lund, Sweden; 2 Department of Internal Medicine, Department of Clinical Sciences, Skåne University Hospital, Lund University, Malmö, Sweden; 3 Department of Orthopaedics, Department of Clinical Sciences Malmö, Skåne University Hospital, Lund University, Malmö, Sweden

**Keywords:** Fracture risk, FRAX, Screening, Women

## Abstract

***Summary*:**

The aim of the study was to evaluate the feasibility and most efficient way of offering middle-aged Swedish women a primary fracture screening program via a questionnaire. Two out of five invited women returned the FRAX questionnaire and those contacted directly by mail were most prone to respond.

**Purpose:**

Osteoporosis and its associated fractures are increasing, and this study aims to explore ways to identify women at an increased risk of fracture using the FRAX® algorithm.

**Methods:**

Three thousand middle-aged women were invited and presented a questionnaire distributed by three different methods–by mail, at routine mammography, or internet-based.

**Results:**

In total, 1120 (37.3%) women responded to the questionnaire and agreed to participate. The response rates for the mail, mammography, and internet-based groups were 39.1%, 35.7%, and 25.2% respectively. Women in the mammography group weighed more, were slightly older than the other women, and also had a higher BMI than women from the mail and internet-based groups. No difference was observed between the groups regarding previous fracture, family history for fracture, current smoking, glucocorticoid use, and alcohol usage. The mammography group had a higher median (interquartile range) major osteoporotic FRAX® score (10.0% (7.8–17.0)) than the mail group (9.7% (7.1–15.0); *p* = 0.005) and the internet-based group (8.7% (6.7–14.0); *p* = 0.001).

**Conclusions:**

Two out of five early postmenopausal women returned the questionnaire and women contacted directly by mail were more prone to respond. Out of the participants, 26.6% had a 10-year fracture risk score ≥ 15% according to the FRAX® algorithm.

## Introduction

Osteoporosis and its subsequent high risk of fragility fractures are a huge and growing problem in the Western world. Scandinavia is highly burdened by high fracture rates with the Swedish hip fracture incidence projected to double from 2002 to 2050 [1]. However, an identification of individuals at high risk of fractures even before the first fractures would be ideal, since preventive measures could be initiated early and personal as well as society costs could be decreased. To identify these high-risk individuals, several fracture risk assessment algorithms are available, e.g. FRAX®, GARVAN, and QFracture. FRAX® is the most studied model [2]. FRAX® is a computer-based algorithm including clinical risk factors which can be applied with and without bone mineral density (BMD) [3]. The large SCOOP study from the UK invited 11,580 women aged between 70 and 85 years of age to participate in a screening program utilising the FRAX® risk score as an indicator of increased fracture risk [4] and a decrease in hip fracture risk by 30% was observed in women identified as high risk [5], but it did not affect the overall fracture risk. Another study, the Danish ROSE trial, with the aim to evaluate the effect of primary screening using FRAX includes women between 65 and 80 years of age [6], and results recently published concluded that the systematic screening offered in the ROSE trial had no effect on fracture incidence in all women but a positive effect in women with moderate to high risk of fracture that chose to attend the offered DXA [7]. In 2012, the Swedish National Board of Health and Welfare published guidelines regarding osteoporosis, recommending the use of FRAX® to calculate fracture risk when a clinical suspicion of osteoporosis is present [8]. The cut-off level for consideration of evaluating BMD was set at a major osteoporotic FRAX® risk score ≥ 15% as stated in the guidelines [8].

This study focuses on women since they have a higher fracture risk than men at the same age [9]. Women are also invited to regular Swedish screening programs, i.e. cervical smear and mammography with participation rates of 80 to 82% that could be used as a possibility for further screening. The aim of the study was to evaluate the feasibility of offering women a primary fracture screening program via a well-known standardised questionnaire and to investigate the most efficient way to offer the questionnaire.

## Methods

### Participants

The participants were randomly assigned to one of three different study groups–the mail, internet-based, or mammography group. The target group was women born between January 1, 1951, and September 30, 1960, (56 to 65 years of age) and living in and around the municipality of Lund, Sweden. Using these inclusion criteria, a total of 6128 eligible women were identified by use of the Swedish National Population Registry (“Befolkningsregistret”) on November 11, 2015.

### Study groups

#### Mammography group

The mammography group consisted of 1000 consecutive women planned for a routine mammography. These women received a letter containing an invitation to participate in the study and the study questionnaire in paper version. They were instructed to return the questionnaire to a pre-assigned locked letterbox at the mammography screening centre.

The invitations were sent out approximately 2 weeks prior to the date of the mammography during a 2-month period in 2016. The last returned questionnaire was returned on May 10, 2016. Women attending the local mammography screening centre, but residing in other municipalities around Lund, were also invited to participate in the study if the inclusion criteria are fulfilled.

#### Mail and internet-based groups

From the eligible women, a total of 2000 women were randomly selected and assigned to either the mail or the internet group. In the mail group, the questionnaire was sent in paper version together with an invitation to participate in the study and a prepaid return envelope. In the internet-based group, a letter was sent with a written instruction on how to fill out the algorithm on the computer directly, print it out, and then, return the questionnaire in the prepaid return envelope. The questionnaires for both mail and internet groups were sent on November 13, 2015, and the last answer was returned on May 13, 2016.

#### Non-participants

All women who either did not return the questionnaire or declined to participate by returning the questionnaire with the marked alternative of not wishing to participate are considered non-participants.

### The questionnaire

The questionnaire consisted of the same questions included in the web-based FRAX® algorithm [10]: age (years), weight (kg), height (cm), previous fracture (yes/no), parent fractured hip (yes/no), current smoking (yes/no), glucocorticoids (yes/no), rheumatoid arthritis (yes/no), secondary osteoporosis (yes/no), alcohol 3 or more units per day (yes/no), and BMD if known. Secondary osteoporosis includes, e.g. type I diabetes mellitus, premature menopause, or malabsorption. A missing answer is considered a “no” according to the FRAX® guidelines [11].

The answers of the individual questionnaires were analysed using the Swedish FRAX® algorithm, and major osteoporotic fracture and hip fracture risk scores were calculated. Some questionnaires had no data on body weight and height, and these study participants were by separate letters asked to complete the data, which 27 out of 35 women did.

Two women stated a high body weight (181 and 150 kg). These data were used for the descriptive statistics, but in FRAX, the highest body weight possible to register is 125 kg and was used for the calculations of the FRAX® score. Body mass index (BMI) was calculated as weight (kg) divided by height squared (m^2^) (kg/m^2^).

### Ethical consideration

The study was approved by the Regional Ethical Committee in Lund (2015/349). A returned questionnaire was interpreted as consent to participate in the study. The access to data for women planned for their routine mammography screening was approved by the Deputy Chief Health Officer of the county council (Region Skåne; decision number 175–15).

### Statistical analysis

The distribution of data was assessed using the one-sample Kolmogorov-Smirnov test and all continuous data was non-parametric. Descriptive statistics for all women and for the different groups are presented as median (interquartile range). Differences between the groups were analysed by the independent-sample Kruskal-Wallis test for non-parametric continuous data. Grouped data were assessed by the chi-square test. One-way ANOVA with post hoc Bonferroni correction was used for continuous data and comparisons between the groups. When the percentage of women with different FRAX scores are given, the tests are based on the binomial distribution and the exact confidence intervals (CI) given.

All analyses were two-sided and a *p* value of 0.05 or less was considered statistically significant. The statistical analysis was performed using IBM Statistical Package for Social Sciences (SPSS) version 22.0 (SPSS Inc., Chicago, IL, USA).

## Results

**Fig. 1:**
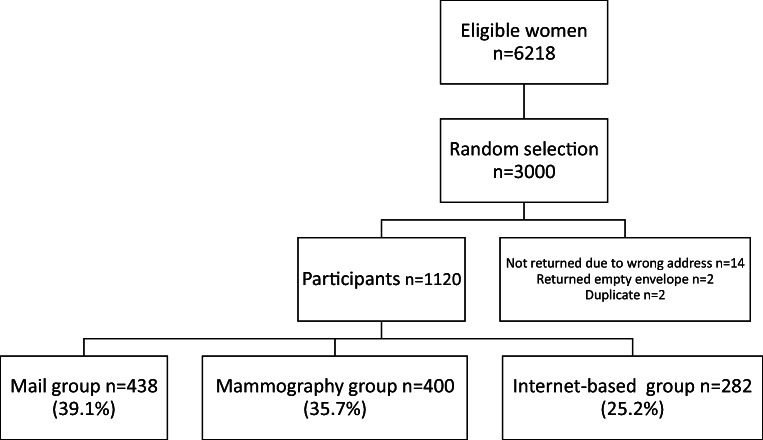
Flowchart for participation in the study

In total, 3000 questionnaires and study invitations were distributed. A total of 1120 accepted to participate in the study (*n* = 37.3%) (Fig. 1). The participation rates for the mail, mammography, and internet-based groups were 39.1%, 35.7%, and 25.2% respectively. The reason for non-participation was optional to fill and the reasons specified were not interested (*n* = 35); feel healthy (*n* = 35); other unspecified (*n* = 28); no access to computer or printer (*n* = 25); did not see the point of calculating FRAX® (*n* = 16), or problems due to language (*n* = 2). Two women received duplicate questionnaires, but only one answer was included in the study.

Women in the mammography group weighed more, were slightly older than the other women, and also had a higher BMI than women from the mail and internet-based groups (Table 1). More women in the mail group stated secondary osteoporosis but fewer with rheumatoid arthritis compared with the mail and internet-based groups (Table 1). No difference was observed between the groups regarding previous fracture, family history for fracture, current smoking, glucocorticoid use, and alcohol usage (Table 1).

**Table 1:** Descriptive data for all women and women in the mammography, mail, and internet-based groups

	All women	Mammography group	Mail group	Internet-based group	
	*n* = 1120	*n* = 400	*n* = 438	*n* = 282	
	Median (IQR^1^)	Median (IQR^1^)	Median (IQR^1^)	Median (IQR^1^)	*p* value^2^
Age (years)	61.0 (58.0–63.0)	62.0 (60.0–64.0)	60.0 (57.0–62.0)	60.0 (57.0–62.0)	*p* < 0.001
Weight (kg)	68.0 (61.0–76.0)	70.0 (62.0–78.0)	67.0 (60.0–77.0)	67.5 (61.0–75.0)	*p* = 0.044
Height (cm)	167.0 (163.0–170.0)	166.0 (162.0–170.0)	167.0 (163.0–171.0)	167.0 (163.0–170.0)	*p* = 0.17
Body mass index (BMI) (kg/m^2^)	24.4 (22.1–27.3)	24.9 (22.6–27.7)	24.0 (21.7–27.1)	24.1 (22.3–26.5)	*p* = 0.004
	*n* (%)		*n* (%)	*n* (%)	
Previous fracture (yes)	221 (19.7)	82 (20.5)	97 (22.1)	42 (14.9)	*p* = 0.13
Parent fractured hip (yes)	230 (20.5)	87 (21.8)	81 (18.5)	62 (22.0)	*p* = 0.49
Current smoking (yes)	89 (7.9)	27 (6.8)	37 (8.4)	25 (8.9)	*p* = 0.64
Glucocorticoids (yes)	94 (8.4)	40 (10.0)	40 (9.1)	14 (5.0)	*p* = 0.093
Rheumatoid arthritis (yes)	49 (8.4)	22 (5.5)	11 (2.5)	16 (5.7)	*p* = 0.004
Secondary osteoporosis (yes)	75 (6.7)	24 (6.0)	40 (9.1)	11 (3.9)	*p* = 0.022
Alcohol 3 or more units per day (yes)	24 (2.1)	5 (1.3)	15 (3.4)	4 (1.4)	*p* = 0.086

^1^Interquartile range

^2^*p* value between mammography, mail, and internet-based groups

As 29 participants in the internet-based group stated that the reason for not participating in the study was due to no access to computer or printer or problems with the internet, they were offered the questionnaire in paper version. A total of 21 women out of the 29 women returned the questionnaire, and these women were still considered participants in the internet-based group.

For all women in the internet-based group, who had filled out the FRAX® algorithm on their computers and returned the questionnaire by mail, the results were checked and 90.9% (239/263) women had managed to use the algorithm correctly. For 19 women, the results were modified due to use of the algorithm for Great Britain, one woman had used the algorithm for the USA, two had not updated the calculation according to their answers, one woman had stated a weight range of 80–85 kg, and one woman had ticked use of corticosteroids but written topical administration on the questionnaire.

In the mammography and mail groups, some women had either marked two response options for the same question (yes and no) or had not marked any answer but instead written a note on the questionnaire. The answers were scrutinised and if difficult to interpret, two of the authors (LM and CB) discussed the answers and reached a consensus. In total, 47 individual answers were changed and for two women, it led to an increased major osteoporotic FRAX® (MO-FRAX® score) (from mean value 11.1 to 17.0) and for 10 women, it decreased the mean MO-FRAX® (from mean 17.4 to 10.3). In the remaining cases, the changes did not affect the cut-off level of 15%. If no conclusive interpretation could be reached, the answer rendering the highest level of risk score was used as to not underestimate the fracture risk. The answers most often changed were those on glucocorticoid use (*n* = 17); rheumatoid arthritis (*n* = 8); secondary osteoporosis (*n* = 8); previous fractures (*n* = 7); hip fracture heredity (*n* = 3); and smoking (*n* = 2).

Of the 1120 women for whom a MO-FRAX® score could be calculated, 298 (26.6% [95% CI 24.0–29.3%]) had MO-FRAX® of ≥ 15% or more (Table 2). Women in the mammography group had higher MO-FRAX® and hip FRAX® scores than women in the mail group and internet-based group, respectively, but no difference was observed between the mail group and internet-based group (Table 2).

**Table 2:** FRAX ®score for all women and for the mammography, mail, and internet-based groups

	All women	Mammography group		Mail group		Internet-based group	
	*n* = 1120	*n* = 400		*n* = 438		*n* = 282	
	Median (IQR^1^)	Median (IQR^1^)	*p* value	Median (IQR^1^)	*p* value	Median (IQR^1^)	*p* value^2^
Major osteoporotic FRAX® score	9.4 (7.3–15.0)	10.0 (7.8–17.0)	*p* = 0.005	9.1 (7.1–15.0)	*p* = 1.00	8.7 (6.7–14.0)	*p* = 0.001
Hip FRAX® score	1.9 (1.2–3.2)	2.2 (1.3–3.4)	*p* = 0.019	1.8 (1.1–3.2)	*p* = 0.66	1.7 (1.2–2.7)	*p* = 0.001
	*n* (%)	*n* (%)		*n* (%)		*n* (%)	
Major osteoporotic FRAX® score ≥ 15%	298 (26.6)	126 (31.5)		113 (25.8)		62 (22.0)	

^1^Interquartile range

^2^*p* value between mammography and internet-based groups

### Non-participants

Some of the non-participating women (*n* = 75) had filled in the questionnaire at the same time as they declined to participate in the study. When comparing non-participants and participants, there was no difference in age, weight, or BMI (data not shown). Non-participants were, however, shorter than participants (*n* = 72; 165.0 (160.0–169.0) cm vs. 167.0 (163.0–170.0) cm; *p* = 0.036). Fewer non-participants than participants had experienced a previous fracture (18.7% vs. 19.7%; *p* = 0.001), reported parents with hip fracture (6.7% vs. 20.5%; *p* = 0.014), or had rheumatoid arthritis (4.0% vs. 4.4%; *p* = 0.018). More non-participants were current smokers (18.7% vs. 7.9%; *p* = 0.006). There were no differences in numbers of glucocorticoid users (10.7% vs. 8.4%; *p* = 0.53), women with secondary osteoporosis (8.0% vs. 6.7%; *p* = 0.18), or women with high alcohol consumption (0% vs. 2.1%; *p* = 0.36). Due to large difference in group size, these results need to be interpreted with caution.

To further evaluate non-participants, 20 of them, randomly chosen, were contacted by phone and queried as to their reasons for not returning the questionnaire. Ten women were from the mammography arm and 10 women were from the mail and internet-based groups. Eight women did not remember the questionnaire at all, and the remaining reasons for not responding were as follows: forgot to bring the questionnaire to the mammography appointment (*n* = 2); changed the location for their mammography and hence, could not return the questionnaire (*n* = 2); did not have the time to respond (*n* = 2); did not feel that the questionnaire was for her (*n* = 2). The remaining four were either away at the time of the questionnaire was delivered (*n* = 1); did not want to participate (*n* = 1); did not have the physical strength due to comorbidity (*n* = 1); and one stated that she had returned the questionnaire, but it had not been registered at the study office (*n* = 1).

## Discussion

In this methodological study, two out of five invited women participated in a screening for osteoporosis. In general, participants had few problems filling out the questionnaire either on the internet or on paper and few had to be contacted to complete the answers. This study compares three different ways to include participants. The mail group is a traditional way of offering a questionnaire whereas the internet-based and mammography groups required some further activity. The mammography group had to bring the questionnaire to the mammography screening centre and when later queried, 2 out of 10 non-participants in the mammography group forgot to do so. The internet-based group had to have access to a computer and printer and also be able follow the instructions on how to fill in the questionnaire on the web page, and this could be one reason why the internet-based group had the lowest participation rate. Sweden does, however, have a high level of access to the internet and a recent survey stated that only 7% of inhabitants between 56 and 66 years of age do not have access to the internet [12].

Swedish women are offered screening for cervical cancer and regular mammography examinations according to national guidelines [13, 14]. A meta-analysis in cervical screening investigating strategies to reach women by offering self-sampling devices showed a response rate between 6.4 and 34.0% (average 19.2%) when a self-sample was mailed to a woman’s home which puts the response rate of this study of 37% in perspective [15]. One of the study arms targeted women attending their regular mammography screening. Women choosing to attend their mammography exam may be more positive towards screening in general, and this could have an impact on the participation rate in the mammography arm. Studies from the nearby city of Malmö observed that non-attendance to the mammography screening was associated with current/previous smoking, teetotalers, strenuous work but little physical activity outside work, vegetarian lifestyle, and a lower self-rated health [16], and also that women who did not attend were more likely to be unmarried without children and also less prone to attend the cervical screening program [17]. Both studies reflect the notion that women with healthy lifestyles are more likely to attend the screening [16, 17]. In this study, however, women in the mammography group were slightly older and heavier, had more sufferers from secondary osteoporosis but otherwise no differences compared with women in the mail and internet-based groups. Hence, mammography women in this study seem not to be different from the remaining women.

Another issue to be addressed is that non-participants may differ from the participants, generating a selection bias. The participants could be healthier than non-participants, but could also suffer from more diseases making them more prone to participate in a study. Among the non-participants of the study, one of the most common reasons for not wanting to participate was that they felt healthy, but none stated that they did not want to participate out of fear of finding out that something might be wrong. Similar findings was concluded from a focus group from the ROSE study that women invited to screening for osteoporosis in general found it acceptable, but a small number of women refused to participate as they felt that their individual risk of osteoporosis was low [18].

The previously mentioned ROSE trial analysed their non-participants and found that they were more likely to be older, to have a lower self-perceived fracture risk, living alone, and being associated to current smoking and alcohol consumption than women who decided to participate [7]. Non-participants in this study did smoke more often than participants and were shorter, but fewer of them had had a previous fracture had a parent with fractured or suffered from RA.

This study has implications for how to plan a screening for increased fracture risk in postmenopausal women. The results of this study show that offering a traditional questionnaire is, in this study, the alternative rendering the most participants, but yet the proportion of women wanting to participate is low. An option is to offer the questionnaire whilst visiting a general practitioner (GP) for other reasons, but that requires a system for notification to the GP.

### Limitations of the study

A limitation of this study is that no reminder was used which may have contributed to lower participation rates. However, since we wanted to compare participation rates between the groups and the women in the mammography group could only return their questionnaires to the mammography centre at their scheduled screening, no reminder was used. The use of a reminder or follow-up in different forms has been shown to increase participation rates [19]; a reminder would probably have increased the participation rate in this study since 8 out of 20 non-participants did not remember the questionnaire at all 6 months after its distribution.

### Conclusion

In conclusion, two out of five invited middle-aged Swedish women returned the screening questionnaire for fracture risk. The highest response rate was in the mail group. Of the responders, 26.6% had a 10-year fracture risk score ≥ 15% according to the FRAX® algorithm. Our results have due implications on how to plan for a primary preventive osteoporosis screening routine in postmenopausal women.
